# Surface decoration of solid lipid nanoparticles with cyclic RGD peptides for precision therapy in high-risk neuroblastoma

**DOI:** 10.1007/s13346-025-01984-9

**Published:** 2025-11-06

**Authors:** Sara Lorenzoni, Carlos Aydillo, Carlos Rodríguez-Nogales, María J. Blanco-Prieto

**Affiliations:** 1 Department of Pharmaceutical Sciences, School of Pharmacy and Nutrition, Universidad de Navarra, Pamplona, Spain; 2 Instituto de Investigación Sanitaria de Navarra, IdiSNA, Pamplona, Spain; 3 Cancer Center Clínica Universidad de Navarra (CCUN), Pamplona, Spain; 4 Department of Pharmaceutics and Food Technology, Faculty of Pharmacy, Complutense University of Madrid, 28040 Madrid, Spain

**Keywords:** Neuroblastoma, Solid lipid nanoparticles, Cyclic RGD, Integrin-targeted therapy, Etoposide, Nanomedicine

## Abstract

High-risk neuroblastoma poses significant therapeutic challenges due to tumor heterogeneity, drug resistance, and systemic toxicity associated with conventional chemotherapies. To overcome these limitations, we developed cyclic RGD-decorated solid lipid nanoparticles for integrin-targeted delivery of etoposide, aiming to enhance tumor selectivity and therapeutic efficacy. SLNs were prepared using hot homogenization and ultrasonication, with cyclic RGD peptides conjugated to the surface via non-covalent and covalent strategies. Among three conjugation approaches evaluated, maleimide-based functionalization was selected for its reproducibility, stability, and high coupling efficiency. RGD-functionalized SLNs were physicochemically characterized and assessed for integrin-mediated uptake, cytotoxicity, cell cycle effects, and apoptosis induction in SH-SY5Y (integrin-high) and SK-N-BE(2) (integrin-low) NB cell lines. RGD-SLNs demonstrated efficient peptide conjugation while maintaining colloidal stability and drug loading. Flow cytometry confirmed enhanced uptake in αvβ3 integrin-expressing SH-SY5Y cells, with moderate uptake in SK-N-BE(2) cells. ETP encapsulation within SLNs significantly improved its cytotoxic profile, with RGD functionalization further reducing IC_50_ values and promoting apoptosis. These findings establish RGD-functionalized SLNs as a promising integrin-targeted platform for ETP delivery in NB. To our knowledge, this is the first report of this approach using SLNs for NB, offering a novel strategy for translational nanomedicine.

**Graphical abstract:**

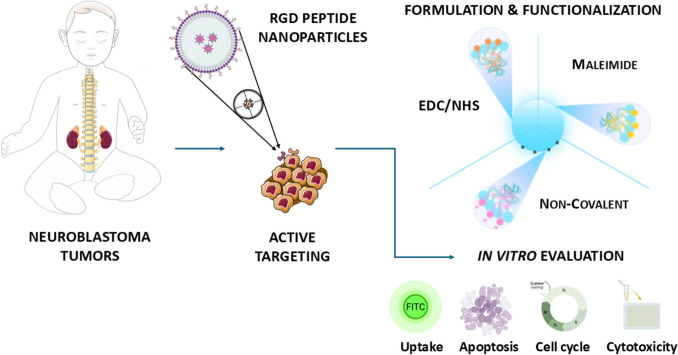

**Supplementary Information:**

The online version contains supplementary material available at 10.1007/s13346-025-01984-9.

## Introduction

Neuroblastoma (NB) is a common extracranial solid tumor in children, accounting for approximately 15% of pediatric cancer-related deaths [1]. Despite advances in multimodal therapies, including surgery, chemotherapy, radiotherapy, and immunotherapy, high-risk NB, particularly in patients with MYCN amplification, remains a significant clinical challenge due to aggressive disease, metastatic potential, drug resistance, and poor prognosis [2, 3]. High-dose chemotherapy, a cornerstone of treatment, often causes severe systemic toxicity, long-term side effects, and drug resistance, highlighting the need for more precise and less toxic therapeutic strategies.

Nanoparticle-based drug delivery systems have emerged as a promising approach to improve drug solubility, stability, and circulation time, thereby enhancing therapeutic index, bioavailability, and safety [4]. However, many systems rely on passive targeting via the enhanced permeability and retention (EPR) effect [5], which can lead to non-specific nanoparticle accumulation and off-target effects [6]. A more specific active tumor targeting can be achieved by functionalizing nanoparticles with specific ligands or antibodies, also exploiting tumor microenvironment (TME)-specific markers, such as receptors or antigens [7]. Peptide-mediated targeting, particularly with arginine-glycine-aspartic acid (RGD) peptides, is advantageous due to its high specificity, biocompatibility, and ease of modification [8]. The αvβ3 integrins, overexpressed in NB cells and critical for angiogenesis, invasion, and metastasis, selectively bind RGD motifs, making them ideal targets for tumor-specific therapies [9, 10].

Lipid nanoparticles are promising drug carriers due to their biocompatibility, tunable surface properties, ability to encapsulate both hydrophilic and hydrophobic drugs, and controlled release capabilities [11, 12]. Among them, solid lipid nanoparticles (SLNs) offer enhanced stability and lower systemic toxicity while efficiently encapsulating lipophilic chemotherapeutic drugs, making them a promising candidate for targeted drug delivery in NB [13–16]. Functionalization of SLNs with monoclonal antibodies [17, 18], peptides [19], or small molecules [20], has been shown to improve specificity and cellular uptake, enhancing antitumor efficacy in preclinical models [21]. RGD-functionalized SLNs can selectively bind αvβ3 integrins, promoting receptor-mediated endocytosis and reducing off-target effects.

In this study, we developed polyethylene glycol (PEG)-coated SLNs functionalized with cyclic RGD (cRGD) peptides to enhance targeted drug delivery in NB by exploiting TME-specific features. We screened multiple functionalization strategies and evaluated three cRGD peptides to optimize targeting efficacy. While linear RGD peptides are well-studied, cRGDs offer superior binding affinity to αvβ3 integrins but remain underexplored [22–25]. Covalent functionalization via carbodiimide (EDC/NHS) [26] and maleimide-thiol coupling [27] alongside non-covalent approaches using electrostatic and hydrophobic interactions, were investigated to achieve stable, site-specific peptide attachment. A comparative analysis of these strategies aimed to optimize SLN affinity for integrin receptors, enhance chemotherapeutic delivery precision, and improve nanoparticle stability in systemic circulation. The chemotherapeutic agent etoposide (ETP), a topoisomerase II inhibitor used in NB treatment [28, 29], was incorporated to leverage its anticancer potential while addressing its limitations, including poor aqueous solubility, rapid clearance, and dose-limiting toxicities. Despite its potent anticancer activity, ETP clinical application is hindered by poor aqueous solubility, rapid systemic clearance, and severe dose-limiting toxicities. In vitro evaluations in SH-SY5Y (integrin-high) and SK-N-BE(2) (integrin-low) NB cell lines, through cell viability, apoptosis, and cell cycle analyses, assessed the safety and therapeutic potential of RGD-functionalized ETP-SLNs, providing mechanistic insights into treatment response. Our cRGD-functionalized, ETP-loaded SLNs aim to improve drug bioavailability and tumor-specific accumulation, enhancing therapeutic efficacy and advancing SLN-based nanocarriers for high-risk NB treatment.

## Experimental section

### Materials

c(RGDfC), c(RGDfK), c(RGDfMeV), and stearic acid-PEG-COOH were acquired from MedChemtronica (Sollentuna, Sweden). Stearic-acid-PEG-Mal was purchased from Telebubio (Barcelona, Spain). Tween80®, etoposide, methanol hypergrade for LC–MS LiChrosolv® and acetonitrile hypergrade for LC–MS LiChrosolv® were purchased from Merck Life Science (Madrid, Spain). Precirol® ATO 5 was a kind gift from Gattefossé (Saint-Priest Cedex, France). Formic acid 99% for mass spectroscopy was obtained from Fluka (Barcelona, Spain). Amicon® Ultra-15 10 kDa MWCO centrifugal filter devices were purchased from Millipore (Cork, Ireland). All reagents for cell culture were acquired from Gibco® (Thermo Fisher Scientific, Waltham, MA, USA) and CellTiter 96® AQueous Non-Radioactive Cell Proliferation Assay (MTS) was purchased from Promega (Madrid, Spain). Annexin V/PI Apoptosis Kit (Molecular Probes) and FxCycle™ PI/RNase Staining Solution (Molecular Probes) were purchased from Thermo Fisher Scientific (Waltham, MA, USA).

### Formulation of solid lipid nanoparticles

SLNs were prepared by the hot homogenization and ultrasonication method [30]. Briefly, the lipid phase, comprising 180 mg of Precirol® ATO 5 and 10 mg of 1,2-dimyristoyl-sn-glycero-3-phosphoethanolamine-N-[methoxy(polyethylene glycol)] (DMG-PEG_2000_), was heated above the melting point of the solid lipids (70 °C). For decorated SLNs, the lipid phase also included 10 mg of a functionalized lipid, as described in the "Characterization of nanoparticles" section and Table S1. For drug-loaded SLNs, ETP (5 mg) was dissolved in 200 µL of methanol and added to the melted lipid phase. For cellular uptake studies, fluorescent SLNs were formulated by incorporating fluorescein (Fl, 0.1% w/w) into the lipid phase, pre-dissolved in 200 µL of methanol. A 10 mL aqueous solution of Tween 80 (2% w/v), preheated to 70 °C, was added to the lipid phase. The mixture was sonicated for 4 min at 13 W using a Microson ultrasonic cell disruptor (Misonix, NY, USA). The resulting SLN suspension was cooled in an ice bath for 15 min and washed three times with ultrapure water by centrifugation (4500 × g, 30 min, 4 °C) using 10 kDa Amicon® centrifugal devices to remove excess surfactant.

To freeze-dry the SLNs, freshly prepared suspensions were mixed with a 10% w/v aqueous trehalose solution as a cryoprotectant and frozen at − 80 °C for at least 12 h. The samples were then lyophilized for 48 h using a Telstar LyoBeta Mini freeze dryer (Syntegon Telstar, SLU, Barcelona, Spain). The lyophilized SLN powders were stored in airtight containers at 4 °C until further use.

### RGD-peptide coupling

**Fig. 1:**
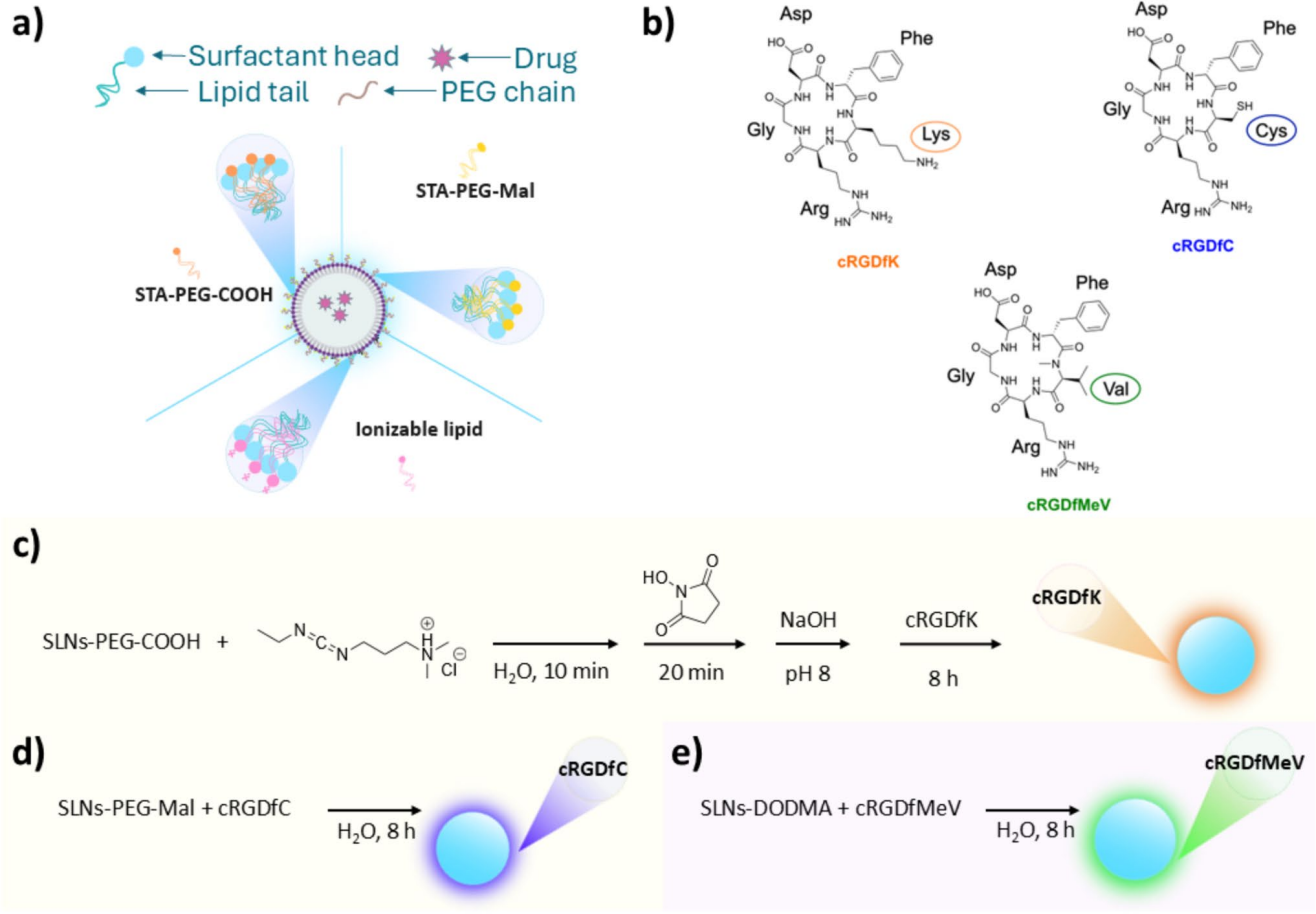
**a)** SLNs functionalization strategies. **b)** cRGD peptides’ structure. **c)** RGD peptides coupling strategies via **(c)** EDC/NHS Chemistry; **(d)** Maleimide-Thiol Chemistry; **(e)** electrostatic functionalization

After optimization, coupling reactions were performed using a 1:2 molar ratio of cRGD to functionalized lipid (Fig. 1a, b), ensuring excess functionalized lipid to promote efficient peptide conjugation while minimizing unreacted peptide. SLN aqueous suspensions were mixed with cRGD and stirred at room temperature for 8 h to facilitate conjugation to their respective functionalized lipids. Purification was achieved by centrifugation (4500 × g, 4 °C, 30 min) using 10 kDa Amicon® centrifugal devices. Detailed methods are described in the following subsections. Functionalization yield was determined by quantifying unbound cRGD via UHPLC-MS/MS, enabling assessment of conjugation efficiency. The percentage of RGD loading on the nanoparticle surface was calculated using the equation:

$$\%Functionalization=\frac{({{mol\;RGD}_0-mol\;RGD}_{supenatant})}{{mol\;RGD}_0}x100$$

#### Non-covalent functionalization

SLNs were formulated with 5% w/w ionizable lipid R-DODMA to enable electrostatic interactions with charged amino acids of the cRGD peptide cilengitide (cRGDfMeV). Briefly, 0.5 µmol of cRGDfMeV was dissolved in 1 mL of ultrapure water and added to a 1 mL aqueous SLN suspension. Immobilization was performed at pH 6.5.

#### Functionalization via EDC/NHS chemistry

SLNs were formulated with 5% w/w STA-PEG2000-COOH. Carboxylic acid residues on the nanoparticle surface were quantified via back titration with NaOH (secondary standard), as detailed in the supporting information (Fig. S1b), revealing 0.74 ± 0.01 µmol/mL of available carboxyl groups. Equimolar amounts of EDC and NHS, each dissolved in 1 mL of ultrapure water, were sequentially added to SLNs within a 10-min interval. The mixture was stirred at room temperature to activate carboxyl groups and form NHS esters. After 20 min, the pH was adjusted to 8.0 using 1 mL of 0.01 M NaOH. Subsequently, 0.37 µmol of cRGDfK was dissolved in 1 mL of ultrapure water and added to the reaction mixture.

#### Functionalization via maleimide-thiol reaction

SLNs were first formulated with 5% w/w of STA-PEG_2000_-Mal. Briefly, 0.5 µmol of cRGDfC was dissolved in 1 mL of ultrapure water and added to a 1 mL aqueous suspension of SLNs at pH 6.0 to form the thioester bond. Additionally, SLNs-STA-PEG_2000_-cRGDfC were prepared using a “one-pot” approach by incorporating cRGDfC directly into the lipid phase during the hot-melting homogenization process. Specifically, the lipid phase comprised 180 mg Precirol® ATO 5, 16 mg DMG-PEG_2000_, 4 mg STA-PEG_2000_-Mal, and 579 μg (1 µmol) cRGDfC.

### Characterization of nanoparticles

Dynamic light scattering (DLS) analyses was performed using a BeNano 180 Zeta Pro analyzer (Bettersize Instruments Ltd., Dandong, China), to determine the average hydrodynamic diameter and polydispersity index (PDI), reported as the mean of three independent measurements. The surface charge, and electrostatic stabilization, of the colloidal systems was evaluated by zeta potential analysis using the same instrument. Surface charge and electrostatic stabilization of the colloidal systems were evaluated by zeta potential analysis using the same instrument. Entrapment efficiency of ETP was quantified using an optimized ultra-high-performance liquid chromatography-tandem mass spectrometry (UHPLC-MS/MS) method previously established by our group [31]. For RGD peptide quantification, a UHPLC-MS/MS method was developed for each peptide. Chromatographic separation was conducted on an Acquity™ Premier BEH C18 VanGuard™ FIT column (2.1 × 50 mm, 1.7 µm; Waters Corp., Milford, MA, USA). Detection was performed using a triple-quadrupole tandem mass spectrometer (Acquity™ TQD, Waters Corp.) with an electrospray ionization (ESI) source operating in positive ion mode. Data acquisition and quantification were managed using MassLynx™ 4.1 software with the QuanLynx™ module (Waters Corp.). Decoration efficiency was calculated as the percentage of peptide successfully conjugated to the SLN surface relative to the initial amount, reported as mean ± standard deviation from three independent experiments.

Fourier-transform infrared (FTIR) spectra were acquired in attenuated total reflectance (ATR) mode using an IR Affinity-1S spectrophotometer (Shimadzu, Japan) equipped with a Specac Golden Gate ATR accessory. Spectra were recorded in the range of 4000–600 cm⁻^1^ with a resolution of 2 cm⁻^1^ and 100 scans per sample.

Proton nuclear magnetic resonance (^1^H NMR) spectroscopy was performed on a Bruker Avance Neo 400 spectrometer (Bruker, Rheinstetten, Germany) operating at 400 MHz. Samples were prepared in a 9:1 mixture of ultrapure water and deuterium oxide (H₂O/D₂O). A standard water suppression pulse sequence (noesygppr1d) was used with 1024 transients to achieve an adequate signal-to-noise ratio. Spectra were recorded at 298 K, with chemical shifts (δ) reported in parts per million (ppm). Spectral data were processed using MestReNova software (Mestrelab Research, Santiago de Compostela, Spain).

### Biological evaluation

cRGD peptides, cRGD-functionalized SLNs and ETP loaded SLNs were evaluated in SH-SY5Y and SK-N-BE(2) NB cell lines purchased from the American Type Culture Collection (Manassas, VI, USA). Cells were cultured in Iscove’s Modified Dulbecco’s Medium (IMDM), supplemented with 10% heat inactivated fetal bovine serum, 1% of insulin-transferrin selenium and 100 U/mL penicillin/100 μg/mL streptomycin. Experiments used cells at passages 3–10 to ensure consistency and minimize phenotypic drift. All reagents for cell culture were sourced from Gibco, Thermo Fisher Scientific Inc., (Waltham, MA, USA). Cells were maintained at 37ºC in a 5% CO_2_ atmosphere.

#### In vitro cytotoxicity

Cell proliferation was assessed using the MTS assay according to the manufacturer’s protocol. Briefly, 5000 cells per well were seeded in 96-well plates and incubated for 24 h. Cells were then treated with serial dilutions of cRGD peptides, cRGD-functionalized SLNs or ETP-loaded SLNs for 72 h. After treatments, 100 µL of MTS reagent (15% v/v) was added per well, followed by a 4 h incubation. Absorbance was measured at 492 nm with a reference wavelength of 690 nm, using a microplate reader (Labsystem, Helsinki, Finland). Dose–response curves were generated to determine the half-maximal inhibitory concentration (IC_50_) by non-linear curve fitting. All experiments were performed in at least three independent replicates.

#### Assessment of nanoparticles uptake

To evaluate the uptake of SLNs, SH-SY5Y and SK-N-BE(2) NB cells were seeded in 6-well plates at a density of 5 × 10^5^ cells per well and incubated overnight at 37ºC. The following day, cells were incubated with Fl-labeled SLNs and cRGD-SLNs at a final concentration of 5 mg/mL for 3 h. After incubation, cells were washed with cold phosphate-buffered saline (PBS, pH 7.4) to remove unbound nanoparticles. Cells were detached by trypsinization, followed by centrifugation at 405 × g for 5 min at 21 °C. The cell pellet was resuspended with 500 µL of PBS supplemented with 0.5% fetal bovine serum (FBS) and 5 mM EDTA and analyzed using a CytoFLEX (DxFLEX) flow cytometer (Beckman Coulter, Barcelona, Spain). Fluorescence from Fl was detected using the 488-nm blue laser and collected through a 525/40 bandpass filter. Mean fluorescence intensity (MFI) values were obtained from 10,000 gated events per sample. Data acquisition and analysis were performed using *CytExpert for DxFLEX*, version 2.2.0.7 software (Beckman Coulter). Appropriate controls were used to set compensation and gating. Results are presented as mean ± standard deviation from three independent experiments.

#### Assessment of apoptosis

Apoptosis was evaluated by dual staining with Annexin V-FITC and propidium iodide (PI) using a commercial apoptosis detection kit (Molecular Probes, Thermo Fisher Scientific Inc., Waltham, MA, USA) according to the manufacturer’s instruction. SH-SY5Y and SK-N-BE(2) NB cells were seeded in 6-well plates at a density of 1.5 × 10^5^ cells per well and incubated overnight at 37 °C in complete growth medium. The following day, cells were incubated with cRGD peptides, cRGD-functionalized SLNs, or ETP-loaded SLNs at their previously determined IC_50_ concentrations for 24 or 72 h. Cells were harvested as described in the "Assessment of nanoparticles uptake" section, washed with cold PBS, and resuspended in Annexin V binding buffer. Annexin V-FITC (5 µL) and PI (1 µL, 100 µg/mL) were added, and samples were incubated for 15 min in the dark on ice. Subsequently, 400 µL of binding buffer was added, and samples were analyzed using a CytoFLEX flow cytometer (Beckman Coulter, Barcelona, Spain) with a 488 nm laser. Annexin V-FITC fluorescence was detected on the FITC channel (525/40 nm bandpass), and PI fluorescence was detected on the PE channel (585/42 nm bandpass). Compensation was applied to correct for spectral overlap between FITC and PI. A total of 10,000 gated events were collected per sample. Data were acquired using CytExpert software and analyzed to distinguish viable, early apoptotic, late apoptotic, and necrotic cells based on FITC and PI signal distribution.

#### Assessment of cell cycle

Cell cycle distribution was determined by PI staining following RNase treatment. Cells were seeded in 6-well plates at a density of 1.5 × 10^5^ cells per well and incubated overnight at 37ºC in complete growth medium. The following day, cells were treated with cRGD peptides, cRGD-functionalized SLNs, or ETP-loaded SLNs at their IC_50_ concentrations for 24 or 72 h. After treatment, cells were harvested, washed with cold PBS, fixed in cold 70% ethanol, and stored at − 20 °C. Fixed cells were washed again with cold PBS and incubated overnight with 500 µL of staining solution containing PI (5 µg/mL) and RNase A (100 µg/mL). DNA content was analyzed using CytoFLEX flow cytometer (Beckman Coulter, Barcelona, Spain) with 488 nm blue laser. PI fluorescence was detected in the PE channel (585/42 nm bandpass), collecting 10,000 events per sample. Cell cycle phases (G0/G1, S, G₂/M) were quantified using *CytExpert for DxFLEX*, version 2.2.0.7 software (Beckman Coulter). The presence of a sub-G1 population was interpreted as indicative of apoptotic DNA fragmentation.

### Statistical analysis

Statistical analyses were performed on data from at least three independent experiments using two-way analysis of variance (ANOVA) followed by Tukey’s post hoc test. Statistical significance was defined as *p ≤ 0.05, **p ≤ 0.01, and ***p ≤ 0.001. Calculations and graphical representations were performed using *OriginPro, Version 2025* (OriginLab Corporation, Northampton, MA, USA).

## Results and discussion

### Synthesis and characterization of RGD-functionalized solid lipid nanoparticles

SLNs were formulated using hot homogenization and ultrasonication method previously described [32], as outlined in the "Formulation of solid lipid nanoparticles" section. For cRGD conjugation, lipid moieties containing a hydrophilic PEG chain with a reactive group for cRGD attachment and a carbonated lipid tail for matrix insertion were incorporated into the SLN surface. Additionally, DMG-PEG_2000_ was included to minimize protein corona formation, enhancing nanoparticle stability in systemic circulation and improving active targeting efficacy [32, 33].

**Fig. 2:**
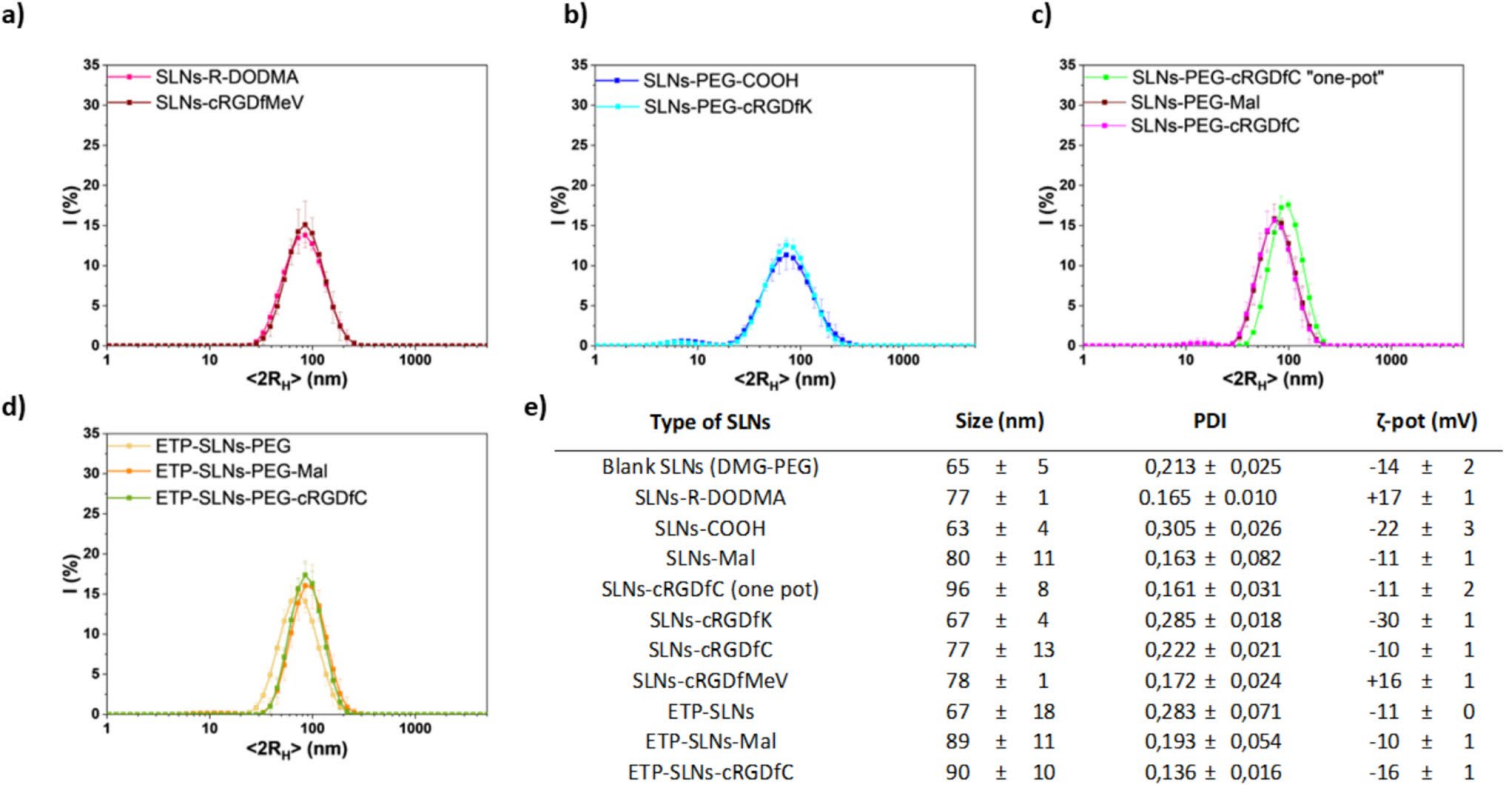
Particle size distribution of SLNs: **(a)** before and after non-covalent coupling, **(b)** before and after EDC/NHS-mediated conjugation, **(c)** before and after maleimide-mediated conjugation, and **(d)** before and after maleimide mediated conjugation of ETP-loaded SLNs. **e)** Summary of hydrodynamic diameter, PDI, and zeta potential values for all formulations. Data are presented as mean ± standard deviation (n = 3)

Blank SLNs, used as a control, showed a mean particle size of 65 ± 5 nm and a zeta potential of −14 ± 2 mV, providing a baseline for comparison with peptide- and drug-loaded formulations. FTIR spectroscopy confirmed the successful incorporation of the PEGylated reactive lipids into the SLNs (Fig. S1 a)). DLS measurements of SLNs before and after RGD peptide conjugation revealed low PDI values (< 0.3) for all formulations, indicating a homogeneous particle size distribution for all the formulations. Particle sizes and zeta potential values remained consistent across formulations (Fig. 2), suggesting that functionalization did not induce particle aggregation or compromise colloidal stability.

Ionizable lipid-based SLNs exhibited a mean hydrodynamic diameter (< 2R_H_ >) of 77 ± 1 nm and a positive zeta potential of + 17 ± 1 mV (Fig. 2(a)). The positive zeta potential indicates partial ionization of the tertiary amine-containing lipid at pH 6.5. Non-covalent immobilization with cRGDfMeV yielded nanoparticles with comparable size and surface charge (ζ ≈ + 16 mV; Fig. 2(a), (e)). The minimal change in ζ-potential suggested low peptide decoration efficiency under these conditions, possibly explained by the cyclic conformation of cRGDfMeV, where the solvent-exposed Arg residue contributes a positive charge that can offset the negative Asp residue. SLNs incorporating carboxylate lipids displayed a smaller size (63 ± 4 nm) and a strongly negative zeta potential of −22 ± 3 mV (Fig. 2(b)), attributed to the presence of ionized carboxyl groups. Following covalent conjugation of cRGDfK via EDC/NHS chemistry, the size increased slightly, while the zeta potential decreased from −22 ± 3 to −30 ± 1 mV (Fig. 2(b, e)), confirming successful peptide attachment. SLNs functionalized with maleimide lipids showed a mean particle size of 80 ± 11 nm and a zeta potential of −11 ± 1 mV (Fig. 2(c)). After conjugation with cRGDfC, the particle size and the zeta potential remained nearly unchanged (Fig. 2(c, e)), confirming that conjugation process did not compromise SLN integrity. However, the SLNs-cRGDfC formulated using the “one-pot” strategy (*i.e.,* incorporating cRGDfC directly into the lipid phase during synthesis) displayed a larger mean particle size (96 ± 8 nm instead of 77 ± 13 nm) but maintained a similar zeta potential of −11 ± 1 mV (Fig. 2(c)), demonstrating the feasibility of simultaneous peptide incorporation during SLN synthesis.

The observed particle sizes (63–96 nm) and PDI values (< 0.3) align with the optimal range of 10–200 nm for active targeting following parenteral administration [34–36]. Particles smaller than 10 nm are prone to rapid renal clearance, leading to reduced systemic availability, while those exceeding 200 nm exhibit limited tissue accumulation via the EPR effect. Moreover, nanoparticles larger than 300 nm are typically cleared by the liver and kidney [37].

### cRGD functionalization of solid lipid nanoparticles

**Fig. 3:**
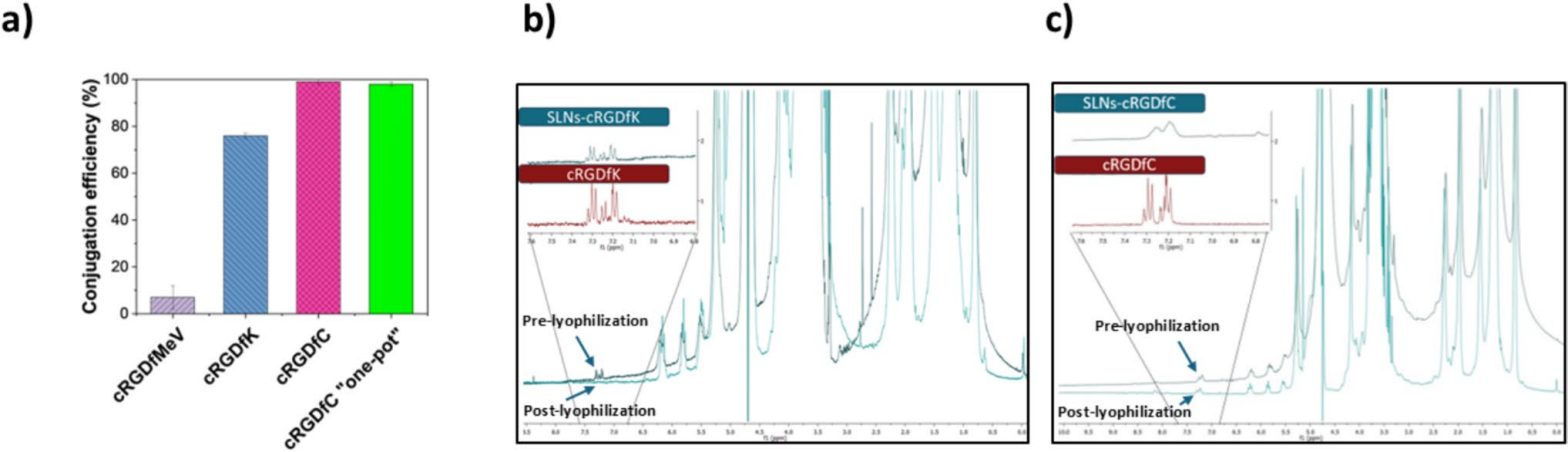
**a)** Conjugation efficiencies of cRGDfMeV, cRGDfK and cRGDfC on SLNs. ^1^H NMR spectra of **(b)** cRGDfK-SLNs and **(c)** cRGDfC-SLNs in H₂O/D₂O (9:1) with solvent suppression. The aromatic region is to compare the free peptide (red), peptide conjugated to SLNs (dark blue), and conjugated peptide after lyophilization (light blue)

First, non-covalent immobilization of cyclic RGD peptide cRGDfMeV onto ionizable SLNs was attempted (Fig. 1(e)). However, UHPLC-MS/MS analysis revealed a low conjugation efficiency of 7 ± 5% (Fig. 3(a)) as observed by UHPLC-MS/MS quantification. Additionally, no detectable NMR signal from the cRGDfMeV peptide was observed on the nanoparticle surface, consistent with poor peptide loading. These findings motivated us to pivot toward covalent conjugation strategies for improved ligand stability and reproducibility. The cRGDfMeV peptide is known to selectively bind to integrins [38], which play key roles in processes such as cell adhesion, migration, and angiogenesis. However, its short circulation half-life limits its in vivo stability and suitability as a targeting moiety [39]. To address this limitation, non-covalent immobilization onto ionizable SLNs was intended to protect cRGDfMeV from nonspecific uptake by cancer cells and rapid degradation or clearance.

To achieve high conjugation efficiency and stable peptide attachment, two covalent coupling strategies were employed (Fig. 1(c, d)): EDC/NHS-mediated amide bond formation for carboxyl-to-amine coupling, and maleimide-thiol chemistry for site-specific thiol conjugation. For this purpose, cRGDfK and cRGDfC were selected as functional analogs of cRGDfMeV, preserving the RGD sequence responsible for integrin-binding specificity. It is important to have in mind that substitution of valine with lysine or cysteine (Fig. 1(b)) does not compromise the biological activity of the ligand, allowing for effective conjugation without loss of targeting capability [40].

SLNs surface-functionalized with cRGDfK, were prepared using EDC/NHS chemistry Fig. 1(c) [28]. Here, the water soluble carbodiimide 1-ethyl-3-(3-dimethylaminopropyl)carbodiimide (EDC) activates the carboxylic acid groups on the SLN surface, forming the unstable *o*-acylisourea intermediate [41]. Upon addition of *N*-hydroxysuccinimide (NHS), it yields a stable NHS-activated ester, which reacts with primary amines to form an amide bond. Surface-exposed carboxylic acid groups were quantified via pH-metric back titration to determine available reactive sites, minimizing side products and enhancing coupling efficiency. Molar ratios of cRGDfK to carboxylic groups (1:10, 1:5, 1:2, 1:1) were tested, yielding conjugation efficiencies of 61, 77, 77, and 74%, respectively. The 1:2 ratio was selected for further studies due to its high yield, consistency, and reproducibility, achieving a conjugation efficiency of 76 ± 1% (Fig. 3(a)).

SLNs functionalized with cRGDfC, were prepared using maleimide-thiol chemistry Fig. 1(d) [29, 42]. Maleimide groups on the SLN surface reacted with the cysteine residue of cRGDfC via sulfa-Michael addition, forming a succinimidyl thioether. The PEGylated lipid fraction was limited to 5% w/w to maintain SLN stability; nevertheless, the optimized maleimide conjugation strategy achieved near-quantitative peptide coupling (99 ± 1%). (Fig. 3(a)), demonstrating high cRGD surface decoration and reproducibility. This result highlights the effectiveness of the proposed reaction in achieving a high conjugation yield with minimal variability. With the aim of reducing reaction times and enhancing the scalability of the formulation process, a one-pot approach was also explored, allowing for the simultaneous incorporation of the cRGD peptide during SLNs formulation. Very interestingly, the one-pot synthesis of SLNs-cRGDfC yielded a comparable conjugation efficiency of 98 ± 1%. One-pot formulations are highly advantageous in pharmaceutical synthesis, offering a streamlined approach to developing formulations [43]. By performing multiple reaction steps in a single vessel without isolating intermediates, this method reduces time and resources, enhancing scalability, and sustainability.

^1^H spectra in deuterated water, recorded before and after lyophilization of RGD-functionalized SLNs, confirmed successful conjugation of cRGDfK and cRGDfC. The characteristic aromatic resonance of the phenylalanine residue in both peptides was observed. For cRGDfK-SLNs, the phenylalanine signal appeared at 7.25–7.40 ppm (Fig. 3(b)), consistent with the free peptide control, indicating preserved structural integrity. However, signal intensity decreased post-lyophilization, suggesting a reduction in the availability of surface-exposed RGD residues. This may result either from detachment of the lipid-PEG-cRGDfK conjugate during sample washing, facilitated by the amphiphilic nature of the peptide, or or from lyophilization-induced nanoparticle aggregation. This highlights a limitation of the EDC/NHS conjugation strategy, which, while forming chemically stable amide linkages, achieves lower functionalization efficiency compared to maleimide-based strategies (Fig. 3(a)). For cRGDfC-SLNs, the phenylalanine signal at 7.15–7.30 ppm (Fig. 3(c)) remained stable after lyophilization, indicating robust peptide attachment via maleimide chemistry. In the one-pot cRGDfC-SLNs, the phenylalanine signal appeared weaker compared to the two-step maleimide strategy (Fig. S2 and Fig. 3(c)). The diminished maleimide signal relative to unreacted maleimide suggests that part of the peptide is exposed at the nanoparticle surface, whereas the broadening of the peptide signals is consistent with partial encapsulation within the ordered SLN core [44]. Such mixed localization may reduce the effective surface presentation of cRGDfC and, consequently, its targeting efficiency.

Various targeting ligands have been explored for NB-specific nanoparticle delivery, including monoclonal antibodies [45, 46], aptamers [47], and small molecules [48]. Monoclonal antibodies offer high specificity but are limited by moderate to high immunogenicity, potential adverse effects, and costly, time-consuming production, hindering clinical translation [49]. Aptamers provide advantages over antibodies, including smaller size, lower immunogenicity, and ease of chemical synthesis and modification [50]. However, their high production costs and limited storage stability restrict broader application [51]. Small-molecule ligands, such as folic acid, offer synthetic flexibility and broad receptor specificity, making them attractive for targeted drug delivery [52]. However, they exhibit lower affinity for tumor-specific binding sites compared to biologically derived ligands. cRGD peptides bridge the gap between small molecules and larger biologics, offering high integrin affinity due to their sequence and three-dimensional conformation [11]. This strong affinity and widespread use for tumor and TME targeting support the rationale for our integrin-targeted SLN system.

### Encapsulation of ETP in cRGD-functionalized solid lipid nanoparticles

To streamline the development process, SLNs functionalized with the cRGDfC peptide (cRGDfC-SLNs) were selected for the encapsulation of ETP and further in vitro biological studies. This choice was supported by the superior stability of cRGD-SLNs, particularly after lyophilization, as confirmed by NMR analysis (Fig. 3(c)). From a manufacturing perspective, the conjugation of cRGDfC to SLNs via a maleimide-thiol reaction presents significant advantages over traditional EDC/NHS coupling. Maleimide chemistry enables site-specific and efficient conjugation under mild aqueous conditions, forming stable thioether linkages that are well suited for scale-up. In contrast, EDC/NHS chemistry is less specific, often requires pH-sensitive activation steps, and may result in heterogeneous conjugation. Given the necessity of lyophilization for long-term storage and formulation stability, the maleimide strategy ensured robust cRGD attachment, preventing peptide detachment or degradation. This stability was critical for ETP encapsulation, as functionalization instability could compromise targeting efficiency and drug retention.

ETP encapsulation led to a slight reduction in particle size, yielding ETP-SLNs with mean hydrodynamic diameter of 67 ± 18 nm (Fig. 2(d)) and a zeta potential of −11 ± 0 mV, indicating strong drug-lipid interactions within the nanoparticle matrix. Further functionalization with maleimide (ETP-SLNs-maleimide) and cRGDfC (ETP-SLNs-cRGDfC) produced particles with diameters of 89 ± 11 nm and 90 ± 10 nm, respectively, with zeta potentials of −10 ± 1 mV and −16 ± 1 mV (Fig. 2(d)). ETP encapsulation efficiencies were 66 ± 4% for ETP-SLNs and 48 ± 1% for ETP-SLNs-cRGDfC, corresponding to lyophilized drug loadings of 3.71 ± 0.30 μg/mg and 2.49 ± 0.29 μg/mg, respectively. Following lyophilization, both ETP-SLNs and ETP-SLNs-cRGDfC exhibited an increase in mean particle size but remained below the 200 nm threshold for intravenous administration. Additional characterization of freeze dried ETP-SLNs and ETP-SLNs-cRGDfC is presented in Fig. S3. These results confirm the successful co-incorporation of ETP and the targeting peptide into SLNs while maintaining nanoscale dimensions and colloidal stability.

### Cellular uptake of cRGD-SLN in neuroblastoma cells

**Fig. 4:**
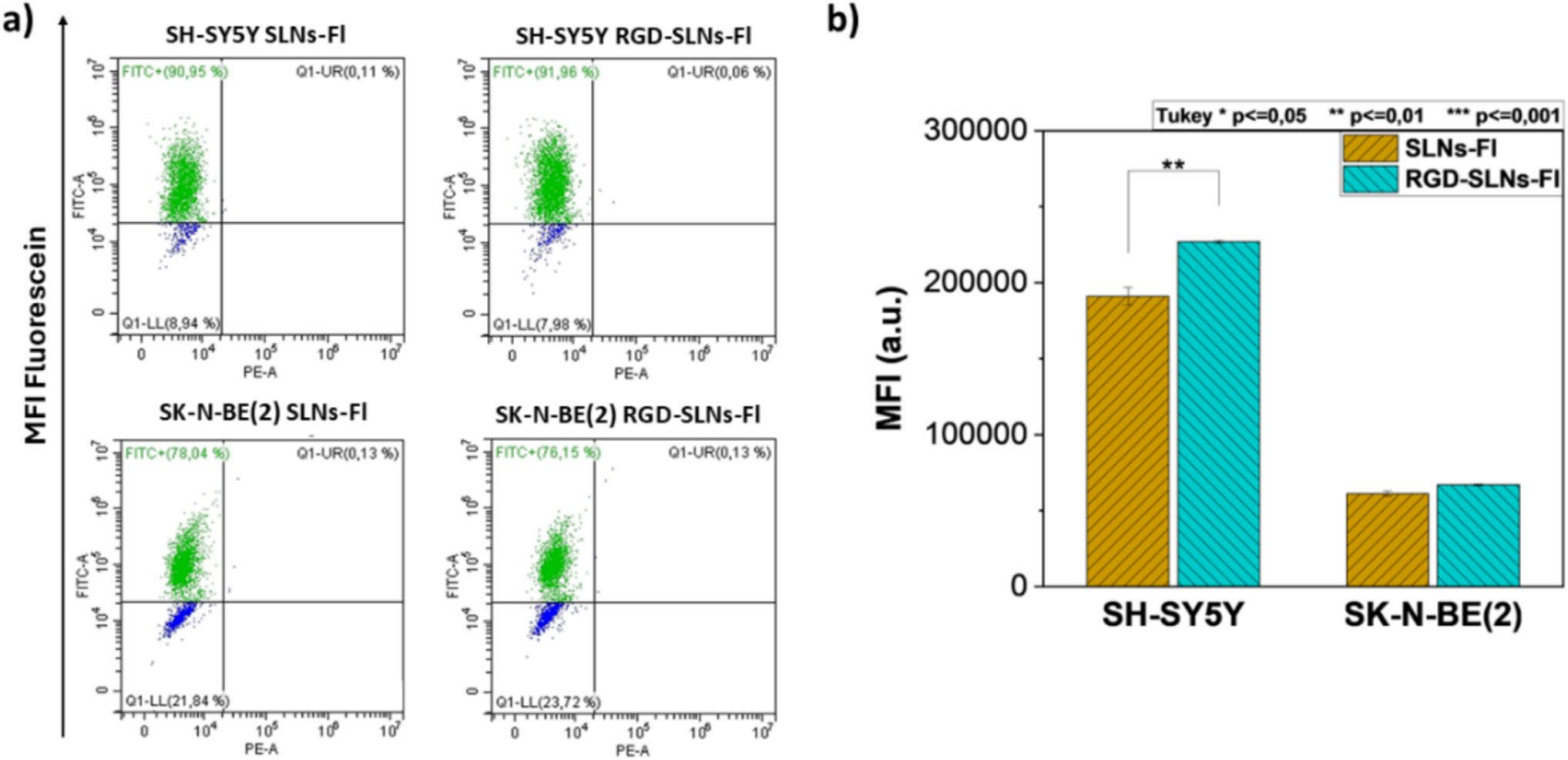
Cellular uptake of fluorescently labelled SLNs in SH-SY5Y and SK-N-BE (2) NB cell lines. **a)** Representative flow cytometry dot plots showing uptake of SLNs-FI and RGD-SLNs-FI after 3 h incubation. The shift in MFI along the Y-axis indicates nanoparticle internalization. **b)** Quantification of cellular uptake expressed as MFI (arbritary units, a.u.). Data are presented as mean ± standard deviation from three independent experiments; *p < = 0.05, **p < = 0.01, ***p < = 0.001 by two-way ANOVA with Tukey post-test

Cellular uptake of fluorescently labeled non-targeted and RGD-decorated SLNs was assessed in SH-SY5Y and SK-N-BE(2) NB cells after 3 h incubation using flow cytometry. Non-targeted Fl-SLNs served as a control for non-specific cellular uptake [53]. As shown in Fig. 4(a), both formulations were internalized, with SH-SY5Y cells exhibiting significantly higher uptake. The mean fluorescence intensity (MFI) in SH-SY5Y cells increased from 190,900 ± 10,120 for Fl-SLNs to 226,691 ± 1,934 for Fl-cRGD-SLNs, indicating enhanced uptake due to cRGD functionalization. In SK-N-BE(2) cells, the MFI increased modestly from 60,953 ± 2489 to 66,733 ± 987, suggesting a less pronounced targeting effect. The MFI increase for Fl-cRGDfC-SLNs in SH-SY5Y cells was statistically significant (*p < 0.01, Tukey’s post hoc test; Fig. 4b). SH-SY5Y cells, which express high levels of αvβ3 integrin (80%) and lack MYCN amplification, serve as an optimal model for integrin-targeted therapies. In contrast, SK-N-BE(2) cells express low levels of αvβ3 integrin (10%) and exhibit MYCN amplification, representing a more aggressive, high-risk NB subtype [16]. These findings align with studies reporting effective targeting of integrin-overexpressing cells mediated by RGD- ligands [54, 55]. This overexpression promotes integrin-mediated endocytosis of RGD-functionalized SLNs, significantly increasing the internalization of nanodrugs by tumor cells [56–58]. These results confirm the successful conjugation of cRGDfC to SLNs via maleimide chemistry ("cRGD functionalization of solid lipid nanoparticles" section) and support the use of cRGD-functionalized SLNs for targeted drug delivery in integrin overexpressing NB models.

### Antitumor efficacy in neuroblastoma cell lines

Cell proliferation was assessed after 72 h treatment to evaluate the intrinsic toxicity of cRGD peptides and SLNs, as well as the therapeutic efficacy of ETP-loaded formulations, using the MTS assay. Free cRGDfK, cRGDfC, and cRGDfMeV peptides exhibited low anticancer activity, with IC_50_ values > 100 µM and cell viability of 70–80%, in SH-SY5Y and SK-N-BE(2) NB cell, indicating limited cytotoxicity (Table 1). To assess baseline toxicity of the nanoparticle carriers, cRGD-functionalized SLNs without ETP were tested at a high concentration of 5 mg/mL. IC_50_ values ranged from 1.06 to 3.23 mg/mL (Table 1), confirming minimal intrinsic cytotoxicity. This screening was essential to define the maximum safe concentrations for subsequent assays with ETP-loaded SLNs. Since the IC_50_ values of the blank RGD-decorated SLNs ranged from 1.06 to 2.67 mg/mL, all cytotoxicity tests with ETP-SLNs were performed below these thresholds. This ensures that the observed cytotoxic effects are attributable to ETP release, rather than any toxicity from the nanoparticle system itself.

**Table 1:** IC_50_ values from MTS assay over 72 hours in SH-SY5Y and SK-N-BE(2) cells

RGD peptides	IC_50_ (μM)	
	SH-SY5Y cell line	SK-N-BE(2) cell line
cRGDfK	>100	>100
cRGDfC	>100	>100
cRGDfMeV	>100	>100
RGD-decorated SLNs	IC_50_ (mg/mL)	
	SH-SY5Y cell line	SK-N-BE(2) cell line
SLNs-cRGDfK	1.51 ± 0.04	1.99 ± 0.60
SLNs-cRGDfC	2.35 ± 0.19	3.23± 0.98
SLNs-cRGDfC “one-pot”	1.16 ± 0.03	2.27 ± 0.04
SLNs-cRGDfMeV	1.34 ± 0.38	1.89 ± 0.24
Ionizable SLNs + cRGDfMeV	1.06 ± 0.09	1.38 ± 0.19
ETP-formulations	IC_50_ (μM)	
	SH-SY5Y cell line	SK-N-BE(2) cell line
Free ETP	0.66 ± 0.10	0.84 ± 0.18
ETP-SLNs	0.25 ± 0.02	0.35 ± 0.01
ETP-SLNs-cRGDfC	0.14 ± 0.01	0.39 ± 0.07

**Fig. 5:**
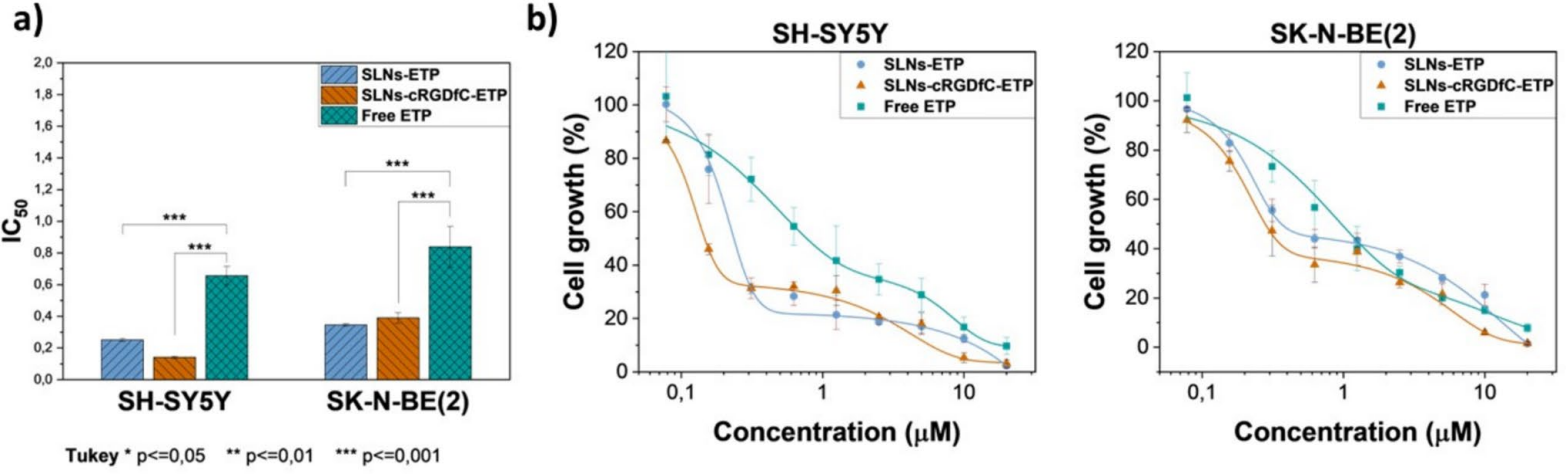
MTS assays in NB SH-SY5Y and SK-N-BE(2) NB cell lines after 72 h treatment with free ETP, ETP-SLNs and cRGD-ETP-SLNs. **a)** quantitative analysis of IC_50_s. Data are presented as mean ± standard deviation from at least three independent experiments; *p < = 0.05, **p < = 0.01, ***p < = 0.001 by two-way ANOVA (Tukey post-test). **b)** Representative dose–response curves

In both cell lines, the cytotoxicity profiles of both non-functionalized and RGD-functionalized ETP-loaded SLNs showed significantly enhanced anticancer efficacy compared to unloaded controls (Table 1). Treatment with ETP-SLNs and ETP-SLNs-cRGDfC induced dose-dependent reductions in cell viability, indicating effective intracellular ETP release. In SH-SY5Y cells, ETP-SLNs-cRGDfC exhibited superior efficacy with an IC_50_ of 0.14 ± 0.01 µM, compared to 0.25 ± 0.02 µM for ETP-SLNs and 0.66 ± 0.10 µM for free ETP (Fig. 5a). This enhanced cytotoxicity correlated with increased cellular uptake of ETP-SLNs-cRGDfC, highlighting the role of integrin-mediated targeting [16]. In SK-N-BE(2) cells, ETP-SLNs-cRGDfC had an IC_50_ of 0.39 ± 0.07 µM, compared to 0.35 ± 0.01 µM for ETP-SLNs and 0.84 ± 0.18 µM for free ETP. These results confirm successful ETP entrapment within the SLN matrix and demonstrate that cRGDfC-mediated targeting enhances intracellular delivery and therapeutic efficacy, particularly in integrin-overexpressing SH-SY5Y cells.

### Apoptosis and cell cycle analysis in neuroblastoma cells

**Fig. 6:**
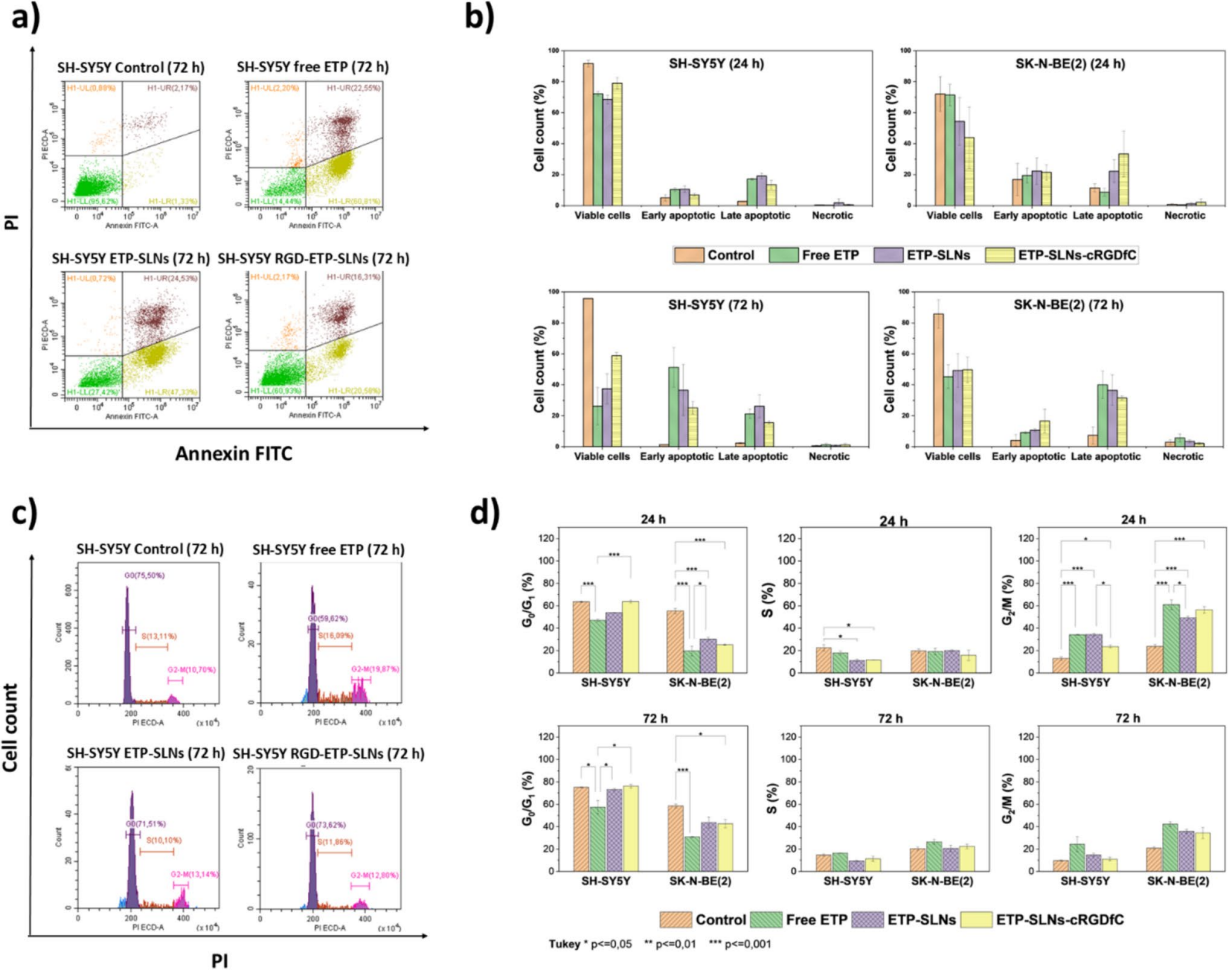
Apoptosis and cell cycle analysis in SH-SY5Y and SK-N-BE(2) NB cell lines after treatment with ETP formulations. (**a**) Representative flow cytometry dot plots of Annexin V-FITC/PI staining in SH-SY5Y cells at 72 h, showing viable (Annexin V⁻/PI⁻), early apoptotic (Annexin V⁺/PI⁻), late apoptotic (Annexin V⁺/PI⁺), and necrotic (Annexin V⁻/PI⁺) populations. (**b**) Quantitative analysis of early and late apoptosis at 24 and 72 h in both cell lines. (**c**) Representative cell cycle histograms at 72 h, determined by PI/RNase staining and flow cytometry. (**d**) Quantitative analysis of G_0_/G₁, S, and G₂/M phase populations. Data are presented as mean ± standard deviation (n = 3); p ≤ 0.05, *p ≤ 0.01, **p ≤ 0.001 by two-way ANOVA with Tukey’s post hoc test

The MTS assay ("Antitumor efficacy in neuroblastoma cell lines" section) provided a measure of metabolic activity and cytotoxicity but could not distinguish between apoptotic and necrotic cell death. Quantitative analysis of apoptosis was performed across both NB cell lines and at different time points (Fig. 6(b)). This analysis confirmed marked differences in response to the formulations. Overall, results indicated minimal necrosis (< 5%), thereby confirming that apoptosis was the dominant mode of cell death. Furthermore, the formulations did not elicit significant nonspecific cytotoxicity, as detailed in Table 1.

At 24 h in SH-SY5Y cells, all ETP-treated groups exhibited increased late apoptosis compared to the control. ETP-SLNs induced the highest late apoptosis (19.07 ± 1.81%), followed by free ETP (17.15 ± 0.60%) and ETP-SLNs-cRGDfC (13.51 ± 2.77%), with no significant differences between these treatments. By 72 h, early apoptosis increased substantially, becoming the primary driver of cell death. ETP inhibits topoisomerase II, causing DNA double-strand breaks and activating the DNA damage response pathway [59]. This initiates early apoptosis, characterized by phosphatidylserine externalization and mitochondrial destabilization, which then progresses to late apoptosis involving nuclear fragmentation and loss of membrane integrity.

Notably, at 24 h in the more chemoresistant SK-N-BE(2) cell line, cRGD-functionalized SLNs led to significantly higher levels of late apoptosis (33.36 ± 14.81%) when compared to non-functionalized ETP-SLNs (22.20 ± 7.45%) and free ETP (8.65 ± 2.53%). This observation was made despite comparable IC_50_ values (0.39 ± 0.07 µM for the targeted formulation vs. 0.35 ± 0.01 µM for the untargeted). We hypothesize that this effect may be partially attributed to enhanced cellular uptake and/or intracellular trafficking facilitated by RGD-integrin interactions. These findings were evident despite the overall reduced nanoparticle uptake compared to SH-SY5Y cells (as previously discussed in the "Cellular uptake of cRGD-SLN in neuroblastoma cells" section).

In cancer cells, mutations in proto-oncogenes and tumor suppressor genes often compromise cell cycle checkpoints, leading to uncontrolled proliferation. Several chemotherapeutic agents, including ETP, exploit this vulnerability by inducing cell cycle arrest, particularly at DNA damage-sensitive checkpoints. To assess the impact of ETP on cell cycle progression, we performed a PI/RNase staining flow cytometry assay. At 24 h post-treatment, all ETP-treated groups in both cell lines exhibited a significant accumulation of cells at the G₂/M phase, indicating checkpoint activation (Fig. 6(d), Table S3). This finding aligns with ETP's known mechanism of action [60]. Representative histograms are shown in Fig. 6(c). In SH-SY5Y, G₂/M population increased from 12.94 ± 2.83% (control) to 34.10 ± 0.79% with free ETP and 34.23 ± 2.09% with ETP-SLNs, confirming that the SLN showed comparable efficacy. However, despite demonstrating enhanced and rapid cellular internalization, cRGDfC-ETP-SLNs elicited a more moderate G₂/M arrest (23.60 ± 2.20%) compared to their non-targeted counterparts. It is important to note that while all formulations were administered at their respective IC_50_ concentrations (determined by MTS assay at 72 h, Table 1), annexin V/PI staining revealed substantial differences in the proportion of viable cells, suggesting distinct modes of cytotoxic action (Table S2) [61].

In SH-SY5Y cells, the percentage of viable cells was 95.72 ± 0.14% for the untreated control, 26.22 ± 12.18% for free ETP, 37.40 ± 9.82% for ETP-SLNs, and 58.80 ± 2.13% for cRGDfC-ETP-SLNs. These findings suggest that, while all treatments were normalized to induce similar metabolic inhibition, the apoptotic assay revealed formulation-dependent variations in cell death mechanisms. This discrepancy highlights the importance of using complementary biological assays to fully capture the complexity of drug-induced responses, especially with nanoparticle-based delivery systems.

SK-N-BE(2) demonstrated a stronger G₂/M response, accompanied by a marked decrease in the G_0_/G₁ population relative to control conditions. This specific shift was not evident in SH-SY5Y (Fig. 6(d), Table S3). By 72 h, the G₂/M accumulation was considerably diminished, suggesting a time-dependent progression toward either repair or apoptosis. This shift was supported by late apoptosis data, as previously discussed. In SH-SY5Y, G₂/M levels dropped to 24.40 ± 11.59% (free ETP), 14.67 ± 2.80% (ETP-SLNs), and 11.05 ± 2.87% (cRGDfC-ETP-SLNs), values close to the control levels (9.69 ± 0.93%). A similar trend was observed in SK-N-BE(2), where the G₂/M population decreased to 42.31 ± 3.74% (free ETP), 35.86 ± 3.23% (ETP-SLNs), and 34.45 ± 8.41% (cRGDfC-ETP-SLNs), relative to 20.88 ± 2.11% in controls.

These findings indicate that ETP-induced G₂/M arrest acts as a priming event, progressively transitioning from checkpoint activation to the initiation of apoptotic pathways. The observed transient G₂/M arrest at 24 h, followed by a return toward baseline at 72 h, aligns with previous reports. For example, Nam et al*.* showed that ETP activates DNA damage response pathways via ATM/p53 in neural progenitor and cancer cells, resulting in checkpoint-mediated accumulation in G₂/M, which is subsequently resolved or leads to cell death [62, 63].

## Conclusions

High-risk NB remains a significant therapeutic challenge, necessitating the development of advanced nanomedicine-based, tumor-targeting strategies. In this study, we screened four peptide conjugation methods and identified maleimide-thiol chemistry as the most efficient and stable for covalently coupling cRGD to SLNs. This approach achieved a conjugation efficiency of 99 ± 1%, yielding nanoparticles with a mean hydrodynamic diameter of < 100 nm and a PDI of < 0.3. Cellular uptake studies demonstrated significantly enhanced internalization of cRGDfC-functionalized SLNs in SH-SY5Y NB cells, which overexpress αvβ3 integrins, compared to non-targeted SLNs. Encapsulation of ETP within these functionalized nanoparticles led to a significantly improved cytotoxic efficacy compared to both free ETP and non-functionalized ETP-SLNs. This enhanced efficacy was characterized by a predominant apoptotic response and minimal necrosis.

These results validate cRGD-functionalized SLNs as an effective platform for integrin-mediated targeted drug delivery in NB models. The synergy of optimized drug release kinetics, enhanced cellular uptake, and stress-induced apoptotic signaling highlights the importance of multimodal screening in nanomedicine development. However, we acknowledge the limitations of 2D in vitro models, as they do not fully recapitulate the TME, including diffusion gradients and extracellular matrix barriers that influence nanoparticle penetration and trafficking. To address these limitations and better predict in vivo outcomes, thereby supporting clinical translation, future studies will utilize 3D tumor spheroid and organoid models to more accurately replicate NB tumor architecture and physiology. By establishing a targeted SLN system, this study significantly advances nanomedicine, positioning cRGD-functionalized nanoparticles as a promising candidate for high-risk NB therapy.

## Supplementary Information

Below is the link to the electronic supplementary material.

Supplementary file1 (DOCX 1.07 MB)

## Data Availability

The datasets generated during the current study are available from the corresponding author on reasonable request. All data generated or analysed during this study are included in this published article and its supplementary information files.

## Abbreviations

*c(RGDfC)*: Cyclo[Arg-Gly-Asp-D-Phe-Cys]
*c(RGDfK)*: Cyclo[Arg-Gly-Asp-D-Phe-Lys]
*c(RGDfMeV)*: Cyclo[Arg-Gly-Asp-D-Phe-*N*(Me)Val], cilengitide
*DLS*: Dynamic light scattering
*EDC*: 1-Ethyl-3-(3-dimethylaminopropyl)carbodiimide
*EPR*: Enhanced permeability and retention
*ETP*: Etoposide
*FBS*: Fetal bovine serum
*Fl*: Fluorescein
*LNPs*: Lipid nanoparticles
*Mal*: Maleimide
*NB*: Neuroblastoma
*NHS*: *N*-Hydroxysuccinimide
*PDI*: Polydispersity index
*PEG*: Polyethylene glycol
*R-DODMA*: 1,2-Dioleyloxy-3-(dimethylamino)propane
*RGD*: Arginine-glycine-aspartic acid motif
*SLNs*: Solid lipid nanoparticles
*STA*: Stearic acid
*TME*: Tumor microenvironment
*UHPLC-MS/MS*: Ultra-high-performance liquid chromatography tandem mass spectrometry
