# Supplementary material for: Surface decoration of solid lipid nanoparticles with cyclic RGD peptides for precision therapy in high-risk neuroblastoma
Source: Drug Deliv Transl Res. 2025 Nov 6;16(7):2316–30. doi: 10.1007/s13346-025-01984-9 (PMC13294253; doi:10.1007/s13346-025-01984-9)
Supplement: Supplementary file 1 — Supplementary file1 (DOCX 1.07 MB) [file 13346_2025_1984_MOESM1_ESM.docx]

**Supporting Information**

**Surface decoration of lipid nanoparticles with RGDs for precision therapy in high-risk neuroblastoma**

Sara Lorenzoni^1,2,3^, Carlos Aydillo^1,2^, Carlos Rodríguez-Nogales^4,*^, María J. Blanco-Prieto^1,2,3,*^

^1^Department of Pharmaceutical Sciences, School of Pharmacy and Nutrition, Universidad de Navarra, Pamplona, Spain.

^2^Instituto de Investigación Sanitaria de Navarra, IdiSNA, Pamplona, Spain.

^3^Cancer Center Clínica Universidad de Navarra (CCUN), Pamplona, Spain.

^4^Department of Pharmaceutics and Food Technology, Faculty of Pharmacy, Complutense University, 28040 Madrid, Spain.

**Corresponding Authors**

* [mjblanco@unav.es](mailto:mjblanco@unav.es), carlor28@ucm.es

**Table S1:**  Summary of nanoparticle formulations

| **Formulation** | **Core components** | | | | **Ligand** | **Functionalization method** | **Conjugation efficiency (%)** |
| --- | --- | --- | --- | --- | --- | --- | --- |
|  | **Precirol® ATO 5** | **DMG-PEG_2000_** | **Reactive lipid** | **Etoposide** |  |  |  |
| Blank SLNs | + | + | N/A | N/A | N/A | N/A | N/A |
| SLNs-R-DODMA | + | + | + | N/A | cRGDfMeV | non-covalent | 7 ± 5% |
| SLNs-COOH | + | + | + | N/A | cRGDfK | EDC/NHS | 76 ± 1% |
| SLNs-Mal | + | + | + | N/A | cRGDfC | maleimide | 99 ± 1% |
| SLNs-cRGDfC (one pot) | + | + | + | N/A | cRGDfC | maleimide | 98 ± 1% |
| ETP-SLNs | + | + | N/A | + | N/A | N/A | N/A |
| ETP-SLNs-Mal | + | + | + | + | cRGDfC | maleimide | 99 ± 1% |

**Characterization of Solid lipid Nanoparticles**

The infrared spectrum of STA-PEG_2000_COOH and SLNs-PEG_2000_COOH is reported in **Fig. S1**. **a)**. Characteristic bands at 2920 and 2855 cm^-1^ correspond to the stretching ν_as_ and ν_s_ vibrations of -CH_2_- groups of aliphatic chain of both Precirol® ATO 5 and STA-PEG_2000_COOH. The intense peak at 1742 cm^-1^ is due to the ν(C=O) absorption of the aliphatic acid ester of Precirol® ATO 5. No peak at 1706 cm^-1^ due to the C=O of COOH was observed, probably due to the overlap with the ester band. Signal at 1472 cm^-1^ is attributed to the bending vibration of -CH_2_- groups. Weak C-O stretching modes of Precirol® ATO 5 occurs in the 1279-1005 cm^-1^ wavenumber region. The absorption at 637 cm^-1^ is assigned to the C-C stretching vibration of the aliphatic chains. Overall, the infrared analysis supports the successful incorporation of STA-PEG_2000_COOH within the SLNs without significant structural alterations, as evidenced by the preserved characteristic peaks of Precirol® ATO 5 and STA-PEG_2000_COOH. The absence of a distinct COOH signal at 1706 cm⁻¹ suggests possible ester interactions or overlap, indicating effective integration of the PEGylated lipid.

To accurately quantify the surface-exposed carboxylic acid groups on functionalized SLNs, a pH-metric back-titration method was employed (**Fig. S1**. **b)**). An excess of sodium hydroxide (NaOH; 10 µmol, 0.010 M, 1000 µL) was added to the SLN suspension (1000 µL), allowing deprotonation of the available –COOH groups. After incubation, the unreacted NaOH was titrated with hydrochloric acid (HCl; 0.00978 M). To correct for background acidity, a blank SLN formulation was analyzed under identical conditions, yielding a baseline acidity of 0.69 µmol/mL. Subtracting this value, the net carboxyl group content attributable to the functionalizable lipid was determined to be 0.74 ± 0.01 µmol/mL. This value was used to optimize EDC/NHS stoichiometry during peptide conjugation, minimizing side-product formation and enhancing coupling efficiency.


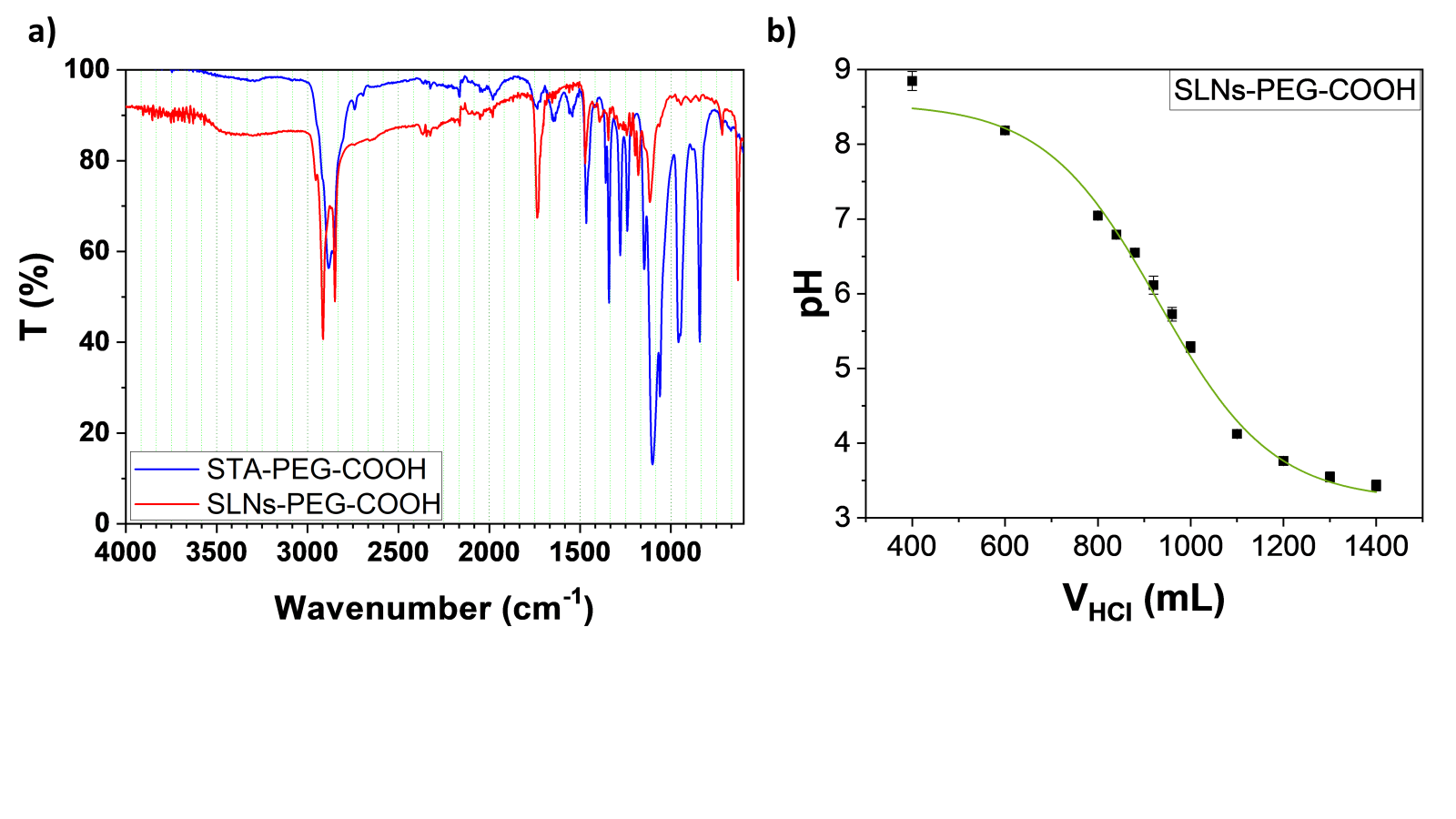


**Fig. S1 a)** pH-metric back titration curve of carboxylic acid residues on the SLN-PEG-COOH surface using a secondary standard NaOH solution. **b)** FTIR spectrum of STA-PEG-COOH and SLNs-PEGCOOH.


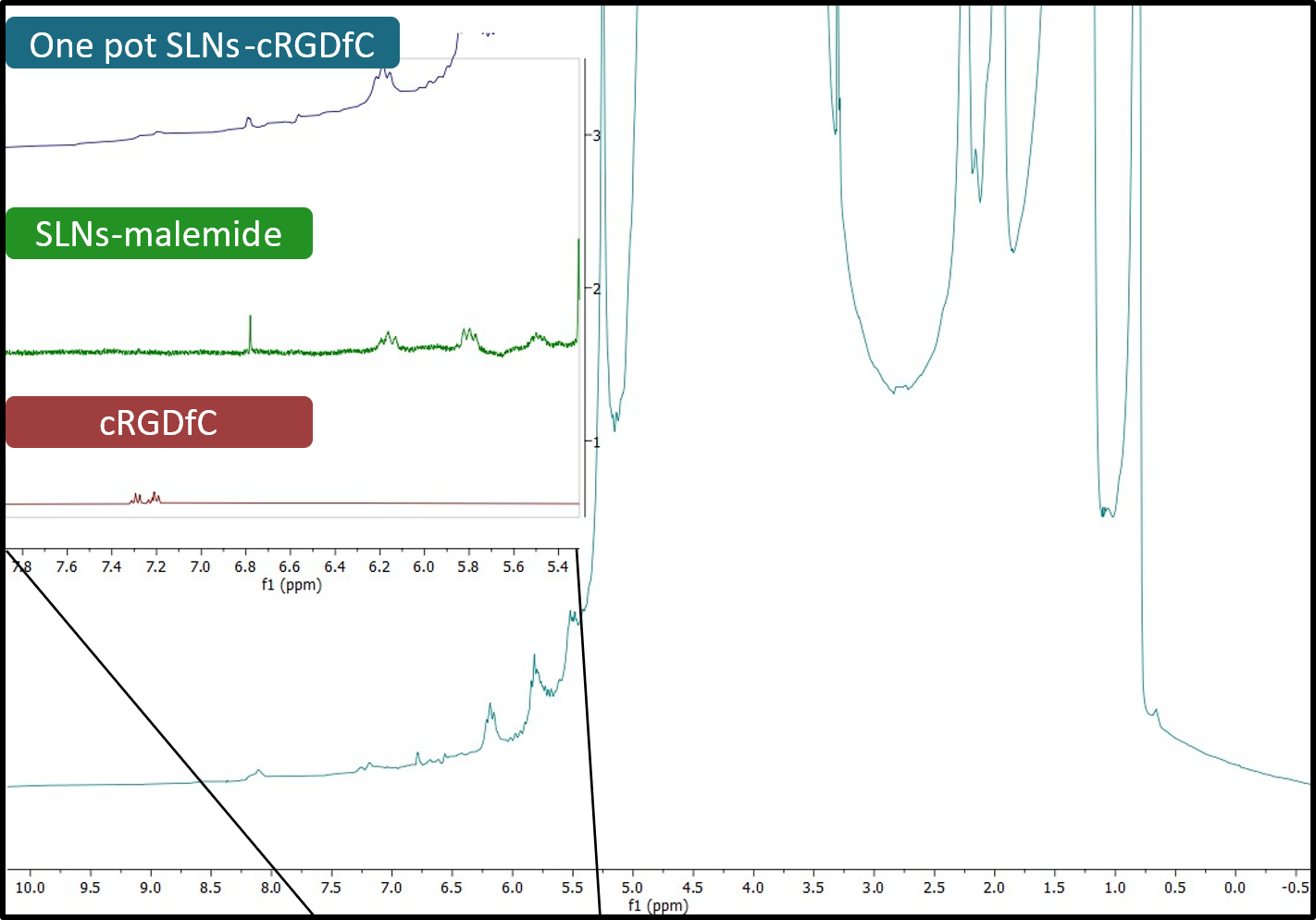


**Fig. S2** ^1^H NMR spectrum of SLNs-cyclic-RGDfC formulated by the “one pot” method in H₂O/D₂O (9:1) with solvent suppression. The aromatic region is amplified to compare the peptide’s signal alone with its signal in the SLNs, and to the signal of SLNs functionalized with unreacted maleimide. The free peptide signal is shown in red, the peptide conjugated to nanoparticles in blue, and the SLNs functionalized with unreacted maleimide in green.


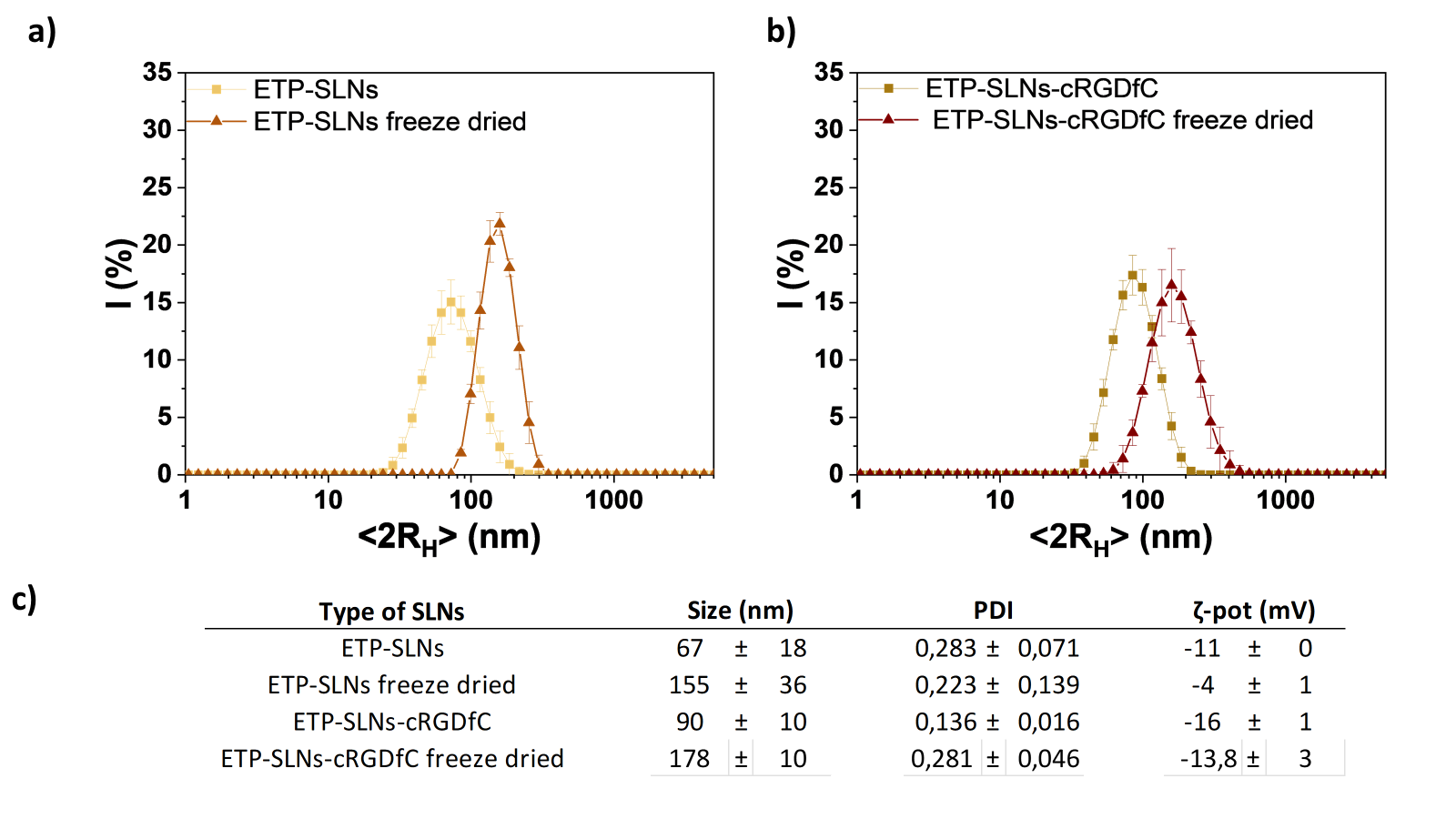


**Fig. S3** Representative particle size distribution profiles of SLNs before and after freeze drying: **(a)** ETP-SLNs, **(b)** EST-SLNs-cRGDfC. **c)** Summary of hydrodynamic diameter, polydispersity index (PDI), and ζ-potential values for all formulations.

**Table S2:**  Annexin V-FITC / Propidium Iodide assay data of 24 h and 72 h treatments in SH-SY5Y and SK-N-BE(2) cell lines.

|  | **24 h treatment** |  | **Viable cells** | | |  | **Early apoptotic cells** | | |  | **Late apoptotic cells** | | |  | **Necrotic cells** | | |
| --- | --- | --- | --- | --- | --- | --- | --- | --- | --- | --- | --- | --- | --- | --- | --- | --- | --- |
| **SH-SY5Y** | Control |  | 91,81 | ± | 2,07 |  | 5,07 | ± | 2,04 |  | 2,75 | ± | 0,23 |  | 0,37 | ± | 0,25 |
|  | Free ETP |  | 72,18 | ± | 1,43 |  | 10,34 | ± | 1,08 |  | 17,15 | ± | 0,60 |  | 0,34 | ± | 0,06 |
|  | ETP-SLNs |  | 68,54 | ± | 2,78 |  | 10,58 | ± | 2,06 |  | 19,07 | ± | 1,81 |  | 1,82 | ± | 2,58 |
|  | cRGDfC-ETP-SLNs |  | 79,02 | ± | 3,63 |  | 6,89 | ± | 0,92 |  | 13,51 | ± | 2,77 |  | 0,57 | ± | 0,34 |
| **SK-N-BE(2)** | Control |  | 72,05 | ± | 11,26 |  | 16,80 | ± | 10,40 |  | 11,42 | ± | 2,61 |  | 0,73 | ± | 0,52 |
|  | Free ETP |  | 71,45 | ± | 7,02 |  | 19,40 | ± | 4,80 |  | 8,65 | ± | 2,53 |  | 0,48 | ± | 0,21 |
|  | ETP-SLNs |  | 54,34 | ± | 15,48 |  | 22,26 | ± | 8,50 |  | 22,20 | ± | 7,45 |  | 1,20 | ± | 0,77 |
|  | cRGDfC-ETP-SLNs |  | 43,91 | ± | 19,84 |  | 21,43 | ± | 4,93 |  | 33,36 | ± | 14,81 |  | 2,14 | ± | 2,22 |
|  |  |  |  |  |  |  |  |  |  |  |  |  |  |  |  |  |  |
|  | **72 h treatment** |  | **Viable cells** | | |  | **Early apoptotic cells** | | |  | **Late apoptotic cells** | | |  | **Necrotic cells** | | |
| **SH-SY5Y** | Control |  | 95,72 | ± | 0,14 |  | 1,39 | ± | 0,08 |  | 2,36 | ± | 0,27 |  | 0,53 | ± | 0,49 |
|  | Free ETP |  | 26,22 | ± | 12,18 |  | 51,30 | ± | 12,76 |  | 21,24 | ± | 2,92 |  | 1,25 | ± | 0,91 |
|  | ETP-SLNs |  | 37,40 | ± | 9,82 |  | 36,57 | ± | 16,50 |  | 26,20 | ± | 7,28 |  | 0,83 | ± | 0,33 |
|  | cRGDfC-ETP-SLNs |  | 58,80 | ± | 2,13 |  | 25,13 | ± | 4,03 |  | 15,60 | ± | 0,95 |  | 1,14 | ± | 0,89 |
| **SK-N-BE(2)** | Control |  | 85,72 | ± | 9,16 |  | 4,15 | ± | 3,75 |  | 7,33 | ± | 5,41 |  | 2,90 | ± | 1,56 |
|  | Free ETP |  | 45,19 | ± | 7,66 |  | 9,14 | ± | 0,67 |  | 40,01 | ± | 8,79 |  | 5,65 | ± | 2,62 |
|  | ETP-SLNs |  | 49,25 | ± | 10,85 |  | 10,72 | ± | 0,88 |  | 36,52 | ± | 9,83 |  | 3,51 | ± | 0,95 |
|  | cRGDfC-ETP-SLNs |  | 49,72 | ± | 8,23 |  | 16,64 | ± | 7,66 |  | 31,48 | ± | 1,34 |  | 2,163 | ± | 0,612 |


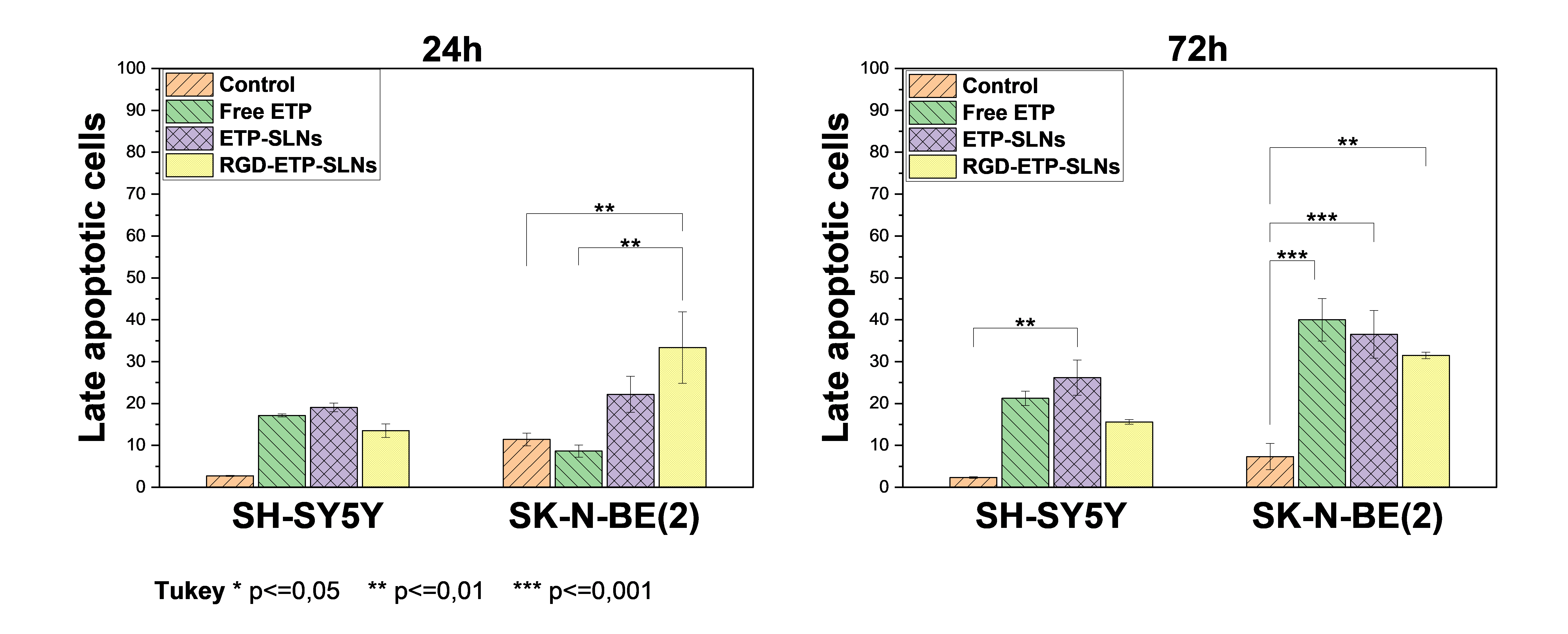


**Fig. S4** Quantitative analysis of late apoptotic cell populations at 24 and 72 hours post-treatment with free ETP, ETP-SLNs, and cRGDfC-ETP-SLNs. Data are presented as mean ± SD (n = 3). Statistical significance was determined by two-way ANOVA followed by Tukey’s post hoc test (*p ≤ 0.05, **p ≤ 0.01, **p ≤ 0.001).

**Table S3:** Cell Cycle Analysis by PI/RNase Staining data of 24 h and 72 h treatments in SH-SY5Y and SK-N-BE(2) cell lines.

|  | **24 h treatment** | | **G0/G1 (%)** | | |  | **S (%)** | | |  | **G2/M (%)** | | |
| --- | --- | --- | --- | --- | --- | --- | --- | --- | --- | --- | --- | --- | --- |
| **SH-SY5Y** | Control |  | 63,63 | ± | 0,74 |  | 22,44 | ± | 4,77 |  | 12,94 | ± | 2,83 |
|  | Free ETP |  | 47,02 | ± | 2,01 |  | 17,95 | ± | 2,76 |  | 34,10 | ± | 0,79 |
|  | ETP-SLNs |  | 53,85 | ± | 0,28 |  | 11,18 | ± | 1,93 |  | 34,23 | ± | 2,09 |
|  | cRGDfC-ETP-SLNs |  | 63,76 | ± | 2,20 |  | 11,70 | ± | 0,26 |  | 23,60 | ± | 2,20 |
| **SK-N-BE(2)** | Control |  | 55,31 | ± | 4,08 |  | 19,69 | ± | 3,07 |  | 23,87 | ± | 2,82 |
|  | Free ETP |  | 19,60 | ± | 7,72 |  | 19,14 | ± | 5,09 |  | 61,05 | ± | 7,65 |
|  | ETP-SLNs |  | 30,19 | ± | 2,95 |  | 19,96 | ± | 1,41 |  | 49,08 | ± | 3,09 |
|  | cRGDfC-ETP-SLNs |  | 25,15 | ± | 0,78 |  | 15,81 | ± | 6,56 |  | 56,40 | ± | 4,19 |
|  |  |  |  |  |  |  |  |  |  |  |  |  |  |
|  | **72 h treatment** | | **G0/G1 (%)** | | |  | **S (%)** | | |  | **G2/M (%)** | | |
| **SH-SY5Y** | Control |  | 75,15 | ± | 0,51 |  | 14,68 | ± | 1,61 |  | 9,69 | ± | 0,93 |
|  | Free ETP |  | 57,42 | ± | 10,53 |  | 16,34 | ± | 0,37 |  | 24,40 | ± | 11,59 |
|  | ETP-SLNs |  | 73,16 | ± | 1,64 |  | 9,32 | ± | 1,13 |  | 14,67 | ± | 2,80 |
|  | cRGDfC-ETP-SLNs |  | 76,21 | ± | 2,90 |  | 11,46 | ± | 3,97 |  | 11,05 | ± | 2,87 |
| **SK-N-BE(2)** | Control |  | 58,56 | ± | 3,31 |  | 20,35 | ± | 2,66 |  | 20,88 | ± | 2,11 |
|  | Free ETP |  | 30,80 | ± | 0,73 |  | 26,58 | ± | 3,56 |  | 42,31 | ± | 3,74 |
|  | ETP-SLNs |  | 43,70 | ± | 8,11 |  | 20,56 | ± | 5,05 |  | 35,86 | ± | 3,23 |
|  | cRGDfC-ETP-SLNs |  | 42,67 | ± | 6,22 |  | 22,38 | ± | 3,38 |  | 34,45 | ± | 8,41 |
